# Diagnostic Accuracy of Body Mass Index and Fasting Glucose for The Prediction of Gestational Diabetes Mellitus after Assisted Reproductive Technology 

**DOI:** 10.22074/ijfs.2019.5505

**Published:** 2019-01-06

**Authors:** Azam Kouhkan, Mohammad E. Khamseh, Ashraf Moini, Reihaneh Pirjani, Arezoo Arabipoor, Zahra Zolfaghari, Roya Hosseini, Hamid Reza Baradaran

**Affiliations:** 1 Endocrine Research Center, Institute of Endocrinology and Metabolism, Iran University of Medical Sciences (IUMS), Tehran, Iran; 2 Department of Endocrinology and Female Infertility, Reproductive Biomedicine Research Center, Royan Institute for Reproductive Biomedicine, ACECR, Tehran, Iran; 3 Department of Gynecology and Obstetrics, Arash Women’s Hospital, Tehran University of Medical Sciences, Tehran, Iran; 4 Vali-e-Asr Reproductive Health Research Center, Tehran University of Medical Sciences, Tehran, Iran; 5 Department of Epidemiology and Reproductive Health, Reproductive Epidemiology Research Center, Royan Institute for Reproductive Biomedicine, ACECR, Tehran, Iran; 6 Department of Andrology, Reproductive Biomedicine Research Center, Royan Institute for Reproductive Biomedicine, ACECR, Tehran, Iran

**Keywords:** Assisted Reproductive Technology, Body Mass Index, Gestational Diabetes Mellitus

## Abstract

**Background:**

The aim of the present study was to determine the maternal pre-pregnancy body mass index (BMI), first-trimester fasting blood sugar (FBS), and the combination of (BMI+FBS) cut-points for at-risk pregnant women conceived by assisted reproductive technology (ART) to better predict the risk of developing gestational diabetes mel- litus (GDM) in infertile women.

**Materials and Methods:**

In this nested case-control study, 270 singleton pregnant women consisted of 135 (GDM) and 135 (non-GDM) who conceived using ART were assessed. The diagnosis of GDM was confirmed by a one-step glucose tolerance test (O-GTT) using 75 g oral glucose. BMI was classified base on World Health Organization (WHO) criteria. The relationship between BMI, FBS, and BMI+FBS with the risk of GDM development was deter- mined by logistic regression and adjusted for confounding factors. Receiver operating characteristic (ROC) curve analysis was performed to assess the value of BMI, FBS, and BMI+FBS for the prediction of GDM.

**Results:**

The GDM group had significantly higher age, BMI, family history of diabetes, and history of polycystic ovary syn- drome in comparison with the non-GDM group (P<0.05). Overweight and obese women had 3.27, and 5.14 folds increase in the odds of developing GDM, respectively. There was a 17% increase in the risk of developing GDM with each 1 mg/dl increase in fasting glucose level. The cut points for FBS 84.5 mg/dl (72.9% sensitivity, 74.4% specificity), BMI 25.4 kg/m^2^ (68.9% sensitivity, 62.8% specificity), and BMI+FBS 111.2 (70.7% sensitivity, 80.6% specificity) was determined.

**Conclusion:**

The early screening and high-quality prenatal care should be recommended upon the co-occurrence of high FBS (≥84.5 mg/dl) in the first-trimester of the pregnancy and the BMI (≥25.4 kg/m^2^) in pre-pregnancy period in women undergone ART. The combination of BMI and FBS is considered a better prediction value.

## Introduction

Gestational diabetes mellitus (GDM) is one of the main obstetrics complications among pregnant women with a history of fertility problem (1), particularly in mothers receiving infertility treatment by assisted reproductive technology (ART) (2). Recent studies reported the association between ART treatment and GDM risk (3). Moreover, pregnancies complicated with GDM can result in adverse maternal and perinatal consequences (4). Genetic predisposition, ethnicity, and age are the most significant risk factors for GDM; furthermore, maternal obesity is consistently proposed as a major and modifiable risk factor (5). Along with obesity accretion rate, there is an increase in the number of obese infertile women seeking infertility treatments through ART (6).

In general terms, GDM is detected at mid-pregnancy (24^th^-28^th^ weeks of gestation) by oral glucose tolerance test (OGTT). Nevertheless, there is evidence suggesting that GDM occurs in all trimesters of pregnancy (7). However, high-risk mothers are assessed in the first-trimester for hyperglycemia in pregnancy. Lately, several studies proposed evaluating the first-trimester prediction of GDM based on maternal characteristics (8). Previously, researchers found that body mass index (BMI) (9) and fasting blood sugar (FBS) level (10, 11) were independent predictors of GDM in normal pregnancy and pregnancies in women with a prior history of polycystic ovary syndrome (PCOS) (12). Recent investigation showed that age, BMI and mode of ART were independent risk factors for GDM in patients undergoing ART (13).

The national institute for health and care excellence (NICE) guidelines (2013) recommended determining the cut-off points for BMI among different populations to help prevent diabetes and the other chronic conditions (14). Furthermore, BMI cut-off as an indicator of GDM was diverse in compliance with race and ethnicity (15). Recent systematic review and meta-analysis evaluated the predictive accuracy of the different combination of GDM risk factors in high-risk women in spontaneous pregnancy (16).

However, there is no consensus about GDM diagnosis regarding screening time, method and, the optimal cut points. Also, there is no direct evidence concerning the cut-off levels for pre-pregnancy BMI and fasting glucose to predict the risk of developing GDM in infertile women as a high-risk population. Accordingly, the present study was designed to evaluate the predictive values of maternal BMI and FBS to predict GDM risk, and then to determine the cut-points for BMI, FBS, and the combination of two biomarkers (BMI+FBS) for the diagnosis of at-risk pregnant women conceived using ART to target clinical surveillance in a more effective manner.

## Materials and Methods

This nested case-control study was conducted between October 2016 and June 2017. The data from 270 women with singleton pregnancies (135 GDM and 135 non-GDM women) conceived by ART treatment referred to Royan Institute were studied. ART was defined as being conceived by intracytoplasmic sperm injection (ICSI) and/ or in vitro fertilization (IVF). Prior to data collection, the protocol of the study was approved by the institutional review board and Ethics Committee of Iran University of Medical Science (Project number: 25469). Clinical records of the participants were reviewed. Consent form was obtained and completed by participants. Data on maternal history and demographic characteristics as well as the records of the first-trimester para-clinical evaluations were collected from the documents. The target population was defined as women with singleton pregnancy via ART and aged between 20-42 years. The exclusion criteria were pre-gestational diabetes; chronic diseases (consisted of hypertension, cardiovascular diseases, untreated thyroid disease, liver diseases, renal diseases, autoimmune diseases, and connective tissue disorders); corticosteroids usage, and incomplete records. Pre-gestational diabetes was defined when the first-trimester FBS was above 125 mg/dl. GDM was confirmed by an OGTT using 75 g oral glucose at the first-trimester (for high-risk subjects) or 24-28 weeks of gestation (for non-GDM subjects). The results of OGTT were interpreted by American diabetes association (ADA) criteria (17). The diagnosis of gestational diabetes was based on FBS =92 mg/dl, 1 hour OGTT =180 mg/dl mg/dl, and 2 hour OGTT =153 mg/dl. Women with high-risk GDM were screened by OGTT on their first antenatal visit. High-risk subjects were defined as individuals with a history of GDM, obesity, impaired glucose metabolism, and history of PCOS. Non-GDM women were screened by OGTT at the 24^th^ to 28^th^ weeks of gestation. Women who had normal OGTT at the 24th to 28^th^ weeks of pregnancy were considered the non-GDM (control) group and individuals with abnormal OGTT were considered the GDM group.

The data pertaining to the characteristics of patients and infertility treatment cycle were collected as previously described in details (18). Pre-pregnancy weight (weight) and height were measured before the initiation of ART cycles by trained nurses. BMI was calculated as the weight in kilograms was divided by the square of height in meters. According to world health organization (WHO), diagnostic criteria (19) women were categorized as normal weight (BMI <25 kg/m^2^), overweight (BMI 25.0-29.9 kg/ m^2^), and obese (BMI=30.0 kg/m^2^).

### Statistical analysis

Data were analyzed using the statistical package for the social sciences (SPSS) software for Windows (version 20, Chicago, IL, USA). Descriptive data were presented as the mean ± standard deviation (SD) or number (%) where appropriate. The independent sample t test was used to compare quantitative data with normal distribution between the two groups. Chi-square test was applied to compare the qualitative variables. The logistic regression analysis was performed to calculate the relationship between BMI, FBS, and BMI+FBS with the risk of GDM after ART cycles. The result of the analysis was expressed as odds ratio (OR) and 95% confidence intervals (CIs). ORs were presented either as crude or adjusted values for confounding variables (age, gravidity, PCOS diagnosis, and family history of diabetes). The patients with BMI <25 kg/m^2^ were considered the reference group. The Hosmer-Lemeshow test was used for the goodness of fit in logistic regression models and the Pearson's chi-square was calculated. The Nagelkerke Pseudo-R2 was determined to quantify predictive ability or model performance.

The receiver operating characteristic (ROC) curve analysis was done by MedCalc statistical software to measure the diagnostic accuracy of BMI, FBS, and BMI+FBS, as well as the optimal cut-point value as predictors for GDM. The DeLong method was used to compare the area under individual and paired ROC curves (AUC). Youden’s index and associated cut-off points were used to measure the overall diagnostic effectiveness. The level of significance was set at P<0.05.

## Results

The clinical and biochemical baseline characteristics of participants are presented in Table 1. The mean maternal age was significantly higher in the GDM group (32.15 ± 5.07 vs. 30.28 ± 4.89, P=0.003). There were significant differences in terms of gravidity, pre-pregnancy weight, BMI, history of diabetes in first relative degree, FBS, and PCOS diagnosis between the two groups (P<0.001 for all variables). There were no significant differences between the two groups in terms of parity, systolic and diastolic blood pressure, maternal education and infertility cause. The incidence of overweight (48.9 vs. 32.5%) and obesity (23.7 vs. 10.9%) was significantly higher in the GDM group (P<0.001).

**Table 1:** Clinical and biochemical baseline characteristics of women conceived via ART with and without GDM


Variable		Non-GDM group n=135	GDM groupn=135	P value

Maternal age (Y)		30.28 ± 4.89	32.15 ± 5.07	0.003
Gravidity (=1, primigravida)		100 (74.0)	79 (58.5)	0.001
Parity (=0, nulliparous)		116 (85.9)	114 (84.4)	0.184
Weight (kg)		64.34 ± 10.18	69.77 ± 10.45	<0.001
BMI (kg/m^2^)		24.57 ± 3.89	27.38 ± 3.91	<0.001
BMI (kg/m^2^)				<0.001
	<25	73 (56.6)	37 (27.4)	
	25.0-29.9	42 (32.5)	66 (48.9)	
	≥30.0	14 (10.9)	32 (23.7)	
History of diabetes in first relative degree		21 (15.5)	62 (45.9)	<0.001
Maternal education				0.636
	Lower secondary	93 ( 68.9)	95 (70.1)	
	Upper secondary	42 (31.1)	40 (29.9)	
FBS (mg/dl)		80.81 ± 5.45	90.66 ± 10.24	<0.001
PCOS diagnosis		11 (8.1)	35 (25.9)	<0.001
Systolic blood pressure (mmHg)		104.26 ± 8.50	106.26 ± 9.77	0.078
Diastolic blood pressure (mmHg)		65.66 ± 7.05	66.70 ± 7.58	0.248
Infertility cause				0.714
	Ovulatory factor	41 (30.3)	48 (35.8)	
	Male factor	65 (48.2)	59 (44.1)	
	Tubal factor	8 (6.0)	9 (6.7)	
	Unexplained	21 (15.5)	18 (13.4)	


Data are presented as mean ± SD or n (%). ART; Assisted reproductive technology, GDM; Gestational diabetes mellitus, BMI; Body mass index, FBS; Fasting blood sugar, and PCOS; Polycystic ovary syndrome.

**Table 2:** Crude and adjusted odds ratios of BMI categories and FBS for development of GDM


Variable		OR crude(95% CI)	OR adjusted(95% CI) (Model 1)	OR adjusted (95% CI)(Model 2)	OR adjusted (95% CI)(Model 3)

BMI ( Kg/m^2^)					
	<25	Reference	Reference	Reference	Reference
	25-29.9	3.10 (1.78,5.39)	2.26 (1.10,4.6)	2.79 (1.37,5.68)	3.27 (1.61,6.66)
	≥30.0	4.51 (2.15,9.47)	2.27 (0.649,7.96)	3.58 (1.05,12.20)	5.14 (1.53,17.26)
Nagelkerke R²		0.118	0.126	0.119	0.116
Hosmer and Lemeshow Test					
	Chi-square	1	2.003	4.08	4.16
	P value^*^	0.01	0.981	0.855	0.842
FBS (mg/dl)		1.171 (1.12-1.20)	1.56 (1.28-1.90)	1.71 (1.41-2.07)	1.4 (1.26-1.56)
Nagelkerke R²		0.364	0.400	0.429	0.422
Hosmer and Lemeshow TestChi-square		15.46	11.87	12.613	12.67
P value^*^		0.051	0.157	0.126	0.124


BMI; Body mass index, FBS; Fasting blood sugar, CI; Confidence interval, GDM; Gestational diabetes mellitus, OR; Odds ratio, and PCOS; Polycystic ovary syndrome. Data are presented as OR (95% CI), Model 1; Adjusted by age and gravidity, Model 2; Adjusted by age, gravidity and PCOS diagnosis, Model 3; Adjusted by age, gravidity, PCOS diagnosis and family history of diabetes, and *; The P value is related to the Hosmer and Lemeshow test- which is not significant- it shows goodness of fitting the model.

Logistic regression analysis illustrated that both FBS level and BMI were significant and independent risk factors for development of GDM after adjustment for confounding variables (age, gravidity, PCOS and family history of diabetes). The results of logistic regression showed that overweight and obese women had a 3.27-fold [adjusted OR (a-OR) 3.27, 95% CI, (1.61, 6.66), P<0.002] and 5.14-fold [aOR 5.14, 95% CI, (1.53, 17.26), P<0.002] higher odds for GDM than that of normal weight women, respectively. There was an approximately 17% increase in the odds of developing GDM with each 1 mg/dl increase in FBS level [OR 1.17, 95% CI, 1.17 (1.12-1.20), P<0.001] (Table 2). 

Our result presents that Nagelkerke Pseudo-R^2^ are 0.118 (BMI) and 0.364 (FBS). It means that our model is stable and can predict the results. The Chi-square of Hosmer- Lemeshow test is the interpreter the goodness of fit test for logistic regression and shows this model is fit for our data.

**Fig.1:**
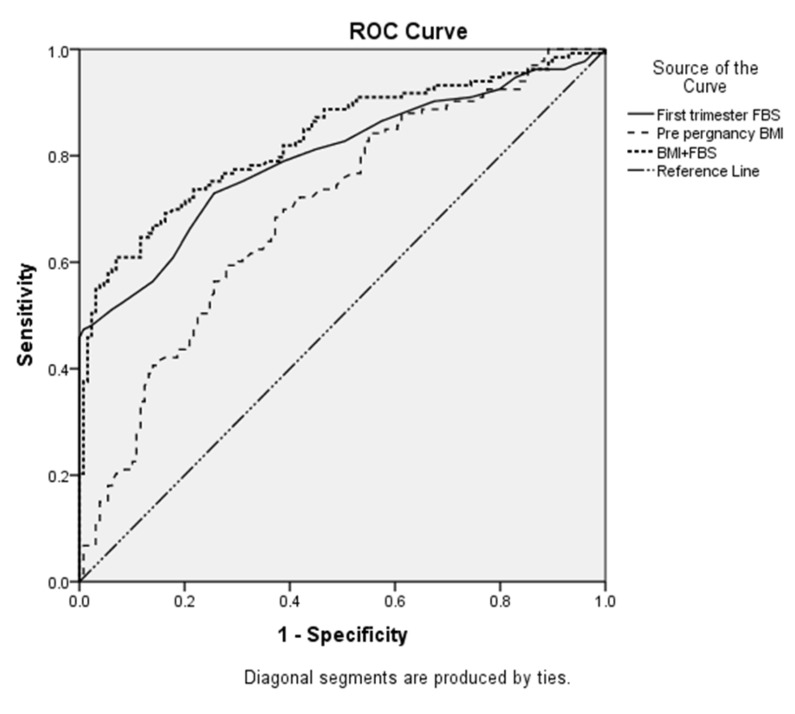
Receiver operating characteristics (ROC) curve analysis for the ability of the first-trimester fasting blood sugar (FBS), pre-pregnancy body mass index (BMI), and BMI+FBS to predict gestational diabetes mellitus (GDM) in women conceived via assisted reproductive technology (ART).

The ROC curves illustrate the ability of FBS, BMI, and BMI+FBS to predict GDM development (Fig .1). The ROC curve analysis showed the predictive values of 0.69, 0.79, and 0.83 for BMI, FBS, and BMI+FBS, respectively.

The values of BMI, FBS, and BMI+FBS for the prediction of GDM and overall diagnostic effectiveness of each factor were presented in Table 3. On the basis of the ROC curves, the best cut-off point for FBS was 84.5 mg/dl, with a sensitivity of 72.9% (95% CI: 64.5-80.3) and specificity of 74.4% (95% CI: 66.0-81.7). Regarding BMI, the best cut-off point was obtained as 25.4 kg/m^2^ with a sensitivity of 68.9% (95% CI: 60.4-76.6), specificity of 62.8% (95% CI: 53.8-71.1). The combination of two biomarkers (BMI+FBS) has a better AUC value (0.83). The best cut- off point for BMI+FBS was 111.2 with a sensitivity of 70.7% (95% CI: 62.2-78.2) and specificity of 80.6% (95% CI: 72.7-87.0), separately. 

**Table 3:** The values of BMI, FBS and BMI+FBS for the prediction of GDM and their overall diagnostic effectiveness


ROC index	BMI	FBS	BMI+FBS

AUC	0.69	0.79	0.83
95% CI of AUC	0.63-0.76	0.74-0.85	0.78-0.88
P value^*^	<0.0001	<0.0001	<0.0001
Youden index J	0.304	0.473	0.513
Cut-off criterion	25.4	84.5	111.2
Sensitivity (%)	68.8	72.9	70.7
95% CI of sensitivity	60.4-76.6	64.5-80.3	62.2-78.2
Specificity	62.79	74.42	80.62
95% CI of specificity	53.8-71.1	66.0-81.7	72.7-87.0
Positive likelihood ratio	1.85	2.85	3.65
Negative likelihood ratio	0.5	0.36	0.36


BMI; Body mass index, FBS; Fasting blood sugar, GDM; Gestational diabetes mellitus, ROC; Receiver operating characteristic, AUC; Under individual ROC curves, CI; Confidence interval, and *; P<0.05 was significant.

The AUC of three ROC curves is compared in Table 4. The results indicate that there are significant differences among pairwise groups. The combination of BMI+FBS significantly improves the predictive ability of FBS or BMI alone for GDM development.

**Table 4:** The pairwise comparison of the area under the ROC curves between BMI, FBS, and BMI+FBS


Variable	BMI vs. FBS	BMI vs. BMI+FBS	FBS vs. BMI+FBS

Difference between areas	0.09	0.13	0.03
Standard error	0.041	0.031	0.013
95% confidence interval	0.016-0.18	0.070-0.19	0.0069-0.060
z statistic	2.35	4.20	2.46
Significance level	P=0.02^*^	P<0.0001^*^	P=0.01^*^


ROC; Receiver operating characteristic, BMI; Body mass index, FBS; Fasting blood sugar, *; P<0.05 was significant.

## Discussion

In the present study, the predictive values of first-trimester FBS, pre-pregnancy BMI, and the combination of two biomarkers for the development of GDM in pregnant women after ART treatment were determined. The results of this study demonstrated that overweight and obese women had approximately 3 and 5 folds increase in the odds of developing GDM, respectively. The cut-off point of 84.5 mg/dl for FBS had a sensitivity of 72.9% and specificity 74.4%, while the cut-off point of 25.4 kg/m^2^ for BMI had a sensitivity of 68.8% and specificity of 62.8%. However, the combination of BMI and FBS significantly improves the predictive ability for GDM development (BMI+FBS cut point: 111.2 with 70.7% sensitivity, 80.6% specificity).

Current evidence indicates that obesity has a negative effect on female reproductive health including ovulatory dysfunction, infertility problems, and poorer outcomes after infertility treatment. Moreover, obesity is associated with impaired ovarian responsiveness to IVF treatment, a lower rate of oocyte fertilization, poor embryo quality, and higher abortion rates (20). 

In our study, a higher incidence of overweight (48.9 vs.32.5%) and obesity (23.7 vs.10.9%) was observed in GDM compared to that of the non-GDM group in the ART population. Provost et al. (21) reported a higher rate of overweight (22.9%) and obesity (17.8%) among women undergoing ART.

Our data show a significant association between BMI and GDM in the ART population. Overweight and obese women had approximately 3 and 5 folds increase in odds of GDM, compared with normal BMI women. Consistent with our findings, Torloni et al. (5), in a meta-analysis of 70 studies, reported that the risk of developing GDM in overweight and obese women in natural pregnancy was almost 2 and 4 folds higher in comparison to normal- weight women. Furthermore, they showed an approximately 0.92% increase in the risk of developing GDM with each 1 kg/m^2^ BMI increase in women with a BMI>25 kg/m^2^, (95% CI: 0.73-1.10). Another study performed by Ogonowski et al. (22), showed that the risk for GDM is increased in parallel with rising in pre-pregnancy BMI not only in overweight but also in normal-weight women. BMI is a strong predictor for GDM requiring insulin therapy. A recent large population-based study revealed the association between BMI and diabetes in pregnancy among women of various ethnicities (23). Moreover, overweight women had a 2.37-fold and obese women had a 5.88-fold increase in the risk of diabetes in pregnancy. Similar to our results, Nishikawa et al. (23) showed that applying a BMI cut-off of 25 kg/m^2^ would identify 68% of South Asian women with diabetes in pregnancy.

Anyway, there is controversy on GDM definition, screening time and method, and threshold values. Previously, a risk factor for GDM was defined as obese women who have BMI above 30 kg/m^2^ (24). ADA indicates BMI 25kg/m^2^ or less as the low-risk group (25). The present study concludes that women with BMI= 25.4 kg/m^2^ are at high risk of GDM development after ART.

Recently, Cai et al. (26) demonstrated that IVF pregnancies are associated with a higher rate of GDM along with elevated fasting and 2-hour OGTT blood glucose levels in the late second-trimester, particularly in overweight and obese mothers; however, the first-trimester FBS was not measured. Previously, Szymanska et al. (27), in a retrospective study, compared 36 singleton pregnant women with GDM who have undergone IVF with 137 non-IVF women with GDM and reported higher levels of FBS in pregnant women with GDM undergone IVF. Similarly, we found that GDM women undergone ART had higher levels of FBS in the first-trimester of pregnancy compared with the non-GDM subjects, but FBS levels were observed within the normal range (FBS <92 mg/dl). Conversely, Sacks et al. (28) found that the measurement of FBS at the first-prenatal visit is not an efficient method for screening of GDM in natural pregnancies because of poor specificity (high false positive rate). Our findings show an approximately 17% increase in the risk of developing GDM with each 1 mg/dl increase in FBS level. In addition, we found a cut-off point of 84.5 mg/dl for FBS with sensitivity 73% and specificity 74%. Riskin-Mashiah et al. (10) reported a cut-off point of 79 mg/dl for FBS using 100 g OGTT with sensitivity 80% and specificity 53% for GDM diagnosis in natural pregnancies. In addition, they found that with each 5 mg/dl increase in fasting glucose or 3.5 kg/m^2^ increase in BMI, the risk of developing GDM increases 1.5-fold among young fertile women.

As there is controversy about GDM screening and diagnosis, recent studies focused on the first-trimester prediction of GDM based on the maternal and clinical characteristics (8, 29, 30) or biomarkers (31) during the natural pregnancy. Hence, in order to predict GDM risk, it is suggested considering multi-parametric models according to maternal clinical risk factors and biomarkers in the first- trimester in pregnancy. Our results showed that the combination of two biomarkers (BMI+FBS) had a cut-point of 111.2 with 70.7% sensitivity and 80.6% specificity to predict GDM development. Moreover, the combination of BMI+FBS significantly improved the predictive ability of FBS or BMI alone for GDM development. Similar to our data, Hao and Lin (30) in a retrospective study carried out on 820 Chinese pregnant women who naturally conceived reported that appropriate fasting plasma glucose (FPG) cut-off value for predicting GDM was 4.6 mmol/L (82.8 mg/dl) with a sensitivity of 53.89% and specificity of 70.90%. The BMI cut-off value was 23.5 kg/m^2^ with a sensitivity of 48.50% and specificity of 73.05%. They found that the combination of these two indices could occupy a larger area under the curve for GDM prediction. Therefore, high levels of FBS or BMI in the first-trimester, especially when combined with each other, should be noticed by health care providers to screen GDM in women who conceived using ART.

On the basis of the authors’ knowledge, this is the first report in ART subjects. The current research has some limitations. We did not evaluate infertility-related factors (hormonal and environmental factors), habits, physical activity, pre-pregnancy waist and hip circumferences, and the dietary regimen of the participants.

## Conclusion

Pre-pregnancy BMI and the first-trimester FBS are independent predictors of GDM in pregnant women conceived by ART. The co-occurrence of high FBS and obesity increases the risk of GDM dramatically in pregnant women conceived by ART.
